# Antimicrobial Resistance and Molecular Epidemiology of *Escherichia coli* Causing Bloodstream Infections in Three Hospitals in Shanghai, China

**DOI:** 10.1371/journal.pone.0147740

**Published:** 2016-01-29

**Authors:** Su Wang, Sheng-Yuan Zhao, Shu-Zhen Xiao, Fei-Fei Gu, Qing-Zhong Liu, Jin Tang, Xiao-Kui Guo, Yu-Xing Ni, Li-Zhong Han

**Affiliations:** 1 Department of Clinical Microbiology, Ruijin Hospital, Shanghai Jiao Tong University School of Medicine, Shanghai, China; 2 Department of Clinical Laboratory, Xiangya Hospital, Central South University, Changsha, Hunan Province, China; 3 Department of Clinical Laboratory, Shanghai First People’s Hospital, Shanghai Jiao Tong University School of Medicine, Shanghai, China; 4 Department of Clinical Laboratory, Shanghai Jiao Tong University Affiliated Sixth People’s Hospital, Shanghai, China; 5 Department of Medical Microbiology and Parasitology, Shanghai Jiao Tong University School of Medicine, Shanghai, China; Leibniz-Institute DSMZ, GERMANY

## Abstract

*Escherichia coli* (*E*. *coli*) is one of the most frequent and lethal causes of bloodstream infections (BSIs). We carried out a retrospective multicenter study on antimicrobial resistance and phylogenetic background of clinical *E*. *coli* isolates recovered from bloodstream in three hospitals in Shanghai. *E*. *coli* isolates causing BSIs were consecutively collected between Sept 2013 and Sept 2014. Ninety isolates randomly selected (30 from each hospital) were enrolled in the study. Antimicrobial susceptibility testing was performed by disk diffusion. PCR was used to detect antimicrobial resistance genes coding for β-lactamases (TEM, CTX-M, OXA, etc.), carbapenemases (IMP, VIM, KPC, NDM-1 and OXA-48), and phylogenetic groups. eBURST was applied for analysis of multi-locus sequence typing (MLST). The resistance rates for penicillins, second-generation cephalosporins, fluoroquinolone and tetracyclines were high (>60%). Sixty-one of the 90 (67.8%) strains enrolled produced ESBLs and no carbapenemases were found. Molecular analysis showed that CTX-M-15 (25/61), CTX-M-14 (18/61) and CTX-M-55 (9/61) were the most common ESBLs. Phylogenetic group B2 predominated (43.3%) and exhibited the highest rates of ESBLs production. ST131 (20/90) was the most common sequence type and almost assigned to phylogenetic group B2 (19/20). The following sequence types were ST405 (8/90) and ST69 (5/90). Among 61 ESBL-producers isolates, B2 (26, 42.6%) and ST131 (18, 29.5%) were also the most common phylogenetic group and sequence type. Genetic diversity showed no evidence suggesting a spread of these antimicrobial resistant isolates in the three hospitals. In order to provide more comprehensive and reliable epidemiological information for preventing further dissemination, well-designed and continuous surveillance with more hospitals participating was important.

## Introduction

Bloodstream infections (BSIs) has been associated with major fatality and prolonged hospital stay for a long period[1]. Reports from North America and Europe have ranked it among the top seven causes of death [2]. Notoriously, *Escherichia coli (E*. *coli)* has emerged as the most common causative gram-negative bacterium [3–7], but even worse, the incidence was keep rising. For example, European Antibiotic Resistance Monitoring Network (EARS-Net) has noted an alarming 71% increase of *E*. *coli* BSIs from 2002 to 2009[4]. In China, the proportion of *E*. *coli* from BSIs (EC-BSI) has jumped from 19.8% to 23.0% during 2010–2012 in accordance with statistics from the Ministry of Health National Antimicrobial Resistance Investigation Net (Mohnarin)[8, 9].

Over the past two decades, the increasing prevalence of *E*. *coli* BSIs was in part driven by an increase in antimicrobial resistant isolates[10]. In the same period from 2002 to 2009, the proportion among all *E*. *coli* of *E*. *coli* resistant to third-generation cephalosporins increased significantly from 1.7% to 8% according to EARS-Net[4]. The most important cause was the production of extended-spectrum β-lactamases (ESBLs) and carbapenemases, with versatile hydrolytic capacity against β-lactams[6, 11, 12]. The genes encoding for these acquired enzymes were related to a high potential for dissemination, and the most widespread type was CTX-M [6, 11]. Data from Mohnarin also showed the positive rate of ESBL-producing EC-BSI had increased from 70.2% to 72.6% during 2009–2012[9]. What’s worse, the therapeutic treatment and cost of *E*. *coli* BSIs has been threatened due to the resistant organisms [13, 14].

In our area, one study has investigated the distribution of pathogens causing BSIs in 2012, and found *E*. *coli* as the most common microorganism accounting for 20% of all the episodes. In addition, CTX-M-14 and ST131was the most common ESBL and sequence type respectively, and phylogenetic group D exhibited the highest rates of ESBL production in one hospital of Shanghai (i.e. Hospital A in this study)[15].Since the former study involving only one hospital was under-represented, and many hospitals didn’t save clinical isolates as a routine work in Shanghai, we chose three hospitals keeping clinical isolates in this present study to outline the same characteristics as above but more comprehensive in the region. This study still sought to determine if the emergence of resistant EC-BSI increased from 2013 to 2014 in Hospital A, as well as to investigate a possible spread of these isolates in and between the hospitals located in three different districts in Shanghai. It was the first multicenter study from these perspectives in Shanghai.

## Materials and Methods

### Study Setting

This retrospective multicenter study was performed in three university-affiliated hospitals located in three different districts[16]: Hospital A(HA) from Huangpu District (1,800 beds), Hospital B(HB) from Xuhui District (1,950 beds) and Hospital C(HC) from Hongkou District (2,350 beds). The distances between HA and HB, HA and HC, HB and HC were 8.9km, 7.6km and 15.0km respectively. All institutions are large-scale general hospitals integrated with emergency, outpatient and inpatient department (including pediatrics, hematology and other basic departments), and medico-technical department. Totally, these hospitals serve a local population of2.6 million approximately representing one fifth of the total population in Shanghai. Apart from native residents, patients from other districts in Shanghai or provinces in China also come to these hospitals for better medical treatment. Besides, these hospitals keep clinical EC-BSI in storage routinely.

### Bacterial Isolates

*E*. *coli*, representing about14.6%(131/898), 18.0%(87/482) and 29.6%(56/189) of all the BSIs episodes in HA, HB and HC respectively, was the most frequent bacilli from BSI in each hospital. A total of 145 *E*. *coli* isolates (58 from HA, 31 from HB, 56 from HC) obtained from blood between 1 Sept 2013 and 30 Sept 2014 were collected. Ninety isolates were enrolled: thirty isolates were selected at random from each hospital using the random number generation function in Microsoft Office Excel2010 (Microsoft Corporation, Redmond, WA, USA). Only nonrepeat isolates from true incident cases were included. All isolates were identified by the VITEK 2 compact system and VITEK 2 Gram-negative identification card (bioMérieux, Marcyl’Étoile, France) and stored at -80°C in broth containing 30% glycerol until used.

This study was approved by Ruijin Hospital Ethics Committee (Shanghai Jiao Tong University School of Medicine), and the Review Board exempted request for informed consent because this retrospective study only focused on the bacteria and did not affect the patients.

### Antimicrobial Susceptibility Testing and Screening and Confirmatory Test for ESBLs and Carbapenemases

Antimicrobial susceptibility profiles of *E*. *coli* isolates were determined by disk diffusion, which included the following drugs: ampicillin, gentamicin, amikacin, piperacillin, ampicillin-sulbactam, piperacillin-tazobactam, cefepime, ceftriaxone, cefuroxime, cefotaxime, ceftazidime, aztreonam, doripenem, imipenem, meropenem, ertapenem, ciprofloxacin, levofloxacin, trimethoprim-sulfamethoxazole, and tetracycline. *E*. *coli* ATCC 25922 and *E*. *coli* ATCC 35218 were used for routine quality control. Throughout this study, results were interpreted using the 2014 CLSI criteria[17]. Screening tests for ESBL and carbapenemase production were performed; double-disk synergy test (cefotaxime and ceftazidime disks with and without clavulanic acid) was used as confirmatory test for ESBL producers following 2014 CLSI criteria[17].K. pneumoniae ATCC 700603 was used as positive control for ESBL production.

### Detection of Resistance Genes

Template DNA was prepared as mentioned by Zhao and colleagues[15]. The primer sequences shown in Table 1 were used in our study for *bla*_TEM_, *bla*_SHV_, *bla*_CTX-M(-1,-2,-8,-9,-25 group)_, *bla*_OXA(-1,-2,-10 group)_, *bla*_VEB_, *bla*_GES_,*bla*_PER_, *bla*_VIM_, *bla*_IPM_, *bla*_KPC_, *bla*_OXA-48_ and *bla*_NDM-1_ genes encoding different β-lactamases and carbapenemases[15, 18]. PCR and sequencing of PCR products were carried out. All PCR fragments were bi-directional sequenced, and the types of β-lactamase genes were identified by comparing the sequences in GenBank (http://www.ncbi.nlm.nih.gov/BLAST).

**Table 1:** Sequences of primers for resistance genes PCR amplification.

Genes	Primers^a^	Primer Sequences (5’-3’)	Expected Amplicon Size (bp)
TEM	TEM F	ATAAAATTCTTGAAGACGAAA	1080
	TEM R	GACAGTTACCAATGCTTAATC	
SHV	SHV F	TGGTTATGCGTTATATTCGCC	865
	SHV R	GGTTAGCGTTGCCAGTGCT	
CTX-M-1^b^	CTX-1 F	AAAAATCACTGCGCCAGTTC	415
	CTX-1 R	AGCTTATTCATCGCCACGTT	
CTX-M-2 ^b^	CTX-2 F	CGACGCTACCCCTGCTATT	552
	CTX-2 R	CCAGCGTCAGATTTTTCAGG	
CTX-M-8 ^b^	CTX-8 F	TCGCGTTAAGCGGATGATGC	666
	CTX-8 R	AACCCACGATGTGGGTAGC	
CTX-M-9 ^b^	CTX-9 F	CAAAGAGAGTGCAACGGATG	205
	CTX-9 R	ATTGGAAAGCGTTCATCACC	
CTX-M-25 ^b^	CTX-25 F	GCACGATGACATTCGGG	327
	CTX-25 R	AACCCACGATGTGGGTAGC	
OXA-1	OXA-1 F	CTGTTGTTTGGGTTTCGCAAG	440
	OXA-1 R	CTTGGCTTTTATGCTTGATG	
OXA-2	OXA-2 F	CAGGCGCYGTTCGYGATGAGTT	233
	OXA-2 R	GCCYTCTATCCAGTAATCGCC	
OXA-10	OXA-10 F	GTCTTTCRAGTACGGCATTA	822
	OXA-10 R	GATTTTCTTAGCGGCAACTTA	
VEB	VEB F	GCGGTAATTTAACCAGA	961
	VEB R	GCCTATGAGCCAGTGTTC	
GES	GES F	ATGCGCTTCATTCACGCAC	846
	GES R	CTATTTGTCCGTGCTCAGG	
PER	PER F	AGTCAGCGGCTTAGATA	978
	PER R	CGTATGAAAAGGACAATC	
KPC	KPC F	TTACTGCCCGTTGACGCCCAATCC	720
	KPC R	TCGCTAAACTCGAACAGG	
IMP	IMP F	AACCAGTTTTGCCTTACCAT	520
	IMP R	CTACCGCAGCAGAGTCTTTG	
VIM	VIM F	TCTACATGACCGCGTCTGTC	953
	VIM R	TGTGCTTTGACAACGTTCGC	
OXA-48	OXA-48 F	ATGCGTGTATTAGCCTTATC	781
	OXA-48 R	CTAGGGAATAATTTTTTCCT	
NDM	NDM F	GCCATGTCACTGAATACTCGT	815
	NDM R	GCGATCCTTCCAACTCGT	
DHA-1,-2^c^	DHA F	AACTTTCACAGGTGTGCTGGGT	405
	DHA R	CCGTACGCATACTGGCTTTGC	
CMY-1, -8 to -11 ^c^	MOX F	GCTGCTCAAGGAGCACAGGAT	520
	MOX R	CACATTGACATAGGTGTGGTGC	
CMY-2 to -7 ^c^	CIT F	TGGCCAGAACTGACAGGCAAA	462
	CIT R	TTTCTCCTGAACGTGGCTGGC	

a Primer, The ‘F’ meant the forward primer and the ‘R’ meant the reverse primer

b CTX-M,Alleles encoding CTX-M enzymes belonging to all five phylogenetic groups. were detected and distinguished by a multiplex PCR assay[18].

c DHA and CMY, the most prevalent plasmid-mediated AmpC worldwide; the primer sequences were consulted in the study of F. J. et al. [23] The PCR would be performed when no ESBL genes were detected in the ESBL-producers, or the ESBL confirmatory test was negative but the screening test was positive. The results were shown in the Discussion.

### Investigations of Genetic Relationship of the Isolates

Multilocus sequence typing (MLST) utilized seven conserved housekeeping genes (*adk*, *fumC*, *gyrB*, *icd*, *mdh*, *purA*, and *recA*) provided by *E*. *coli* MLST database (http://mlst.warwick.ac.uk/mlst/dbs/Ecoli), thus the combination of each allele can define the sequence type for each isolate[19]. eBURST was performed for MLST analysis. The eBURST algorithm groups strains according to their allelic profiles by employing a user-specified group definition, as well as drawing a rough sketch to show the genetic relationship. In this study, strains were grouped together if 6 of the 7 alleles were homologous [20, 21].

*E*. *coli* is composed of four main phylogenetic groups (A, B1, B2, and D). They were determined using a triplex PCR-based method according to the presence of the two genes (chuA and yjaA) and an anonymous DNA fragment(TspE4.C2)[22].

### Statistical Analysis

We presented continuous variables as the mean±SD or median and interquartile range, Categorical variables were evaluated using the chi-square test with Fisher's exact test in the case of small numbers. A two-tailed *p* value of <0.05 was considered statistically significant. All statistical analysis was conducted by SAS 8.2 (SAS Institute Inc., Cary, NC, USA)

## Results

### Characteristics of Total Patient Population

The age of the 90 patients ranged from 2 to 95 years, and the quartile ranged from 52 to 77 years. Males (55/90) are more than females (35/90). Most of the cases (15/90) were derived from the surgery.

### Antimicrobial Resistance Patterns

Sixty-one isolates (67.8%) were ESBLs producers, and carbapenemases producers were not found. Among all isolates, the highest rates of resistance were toampicillin (90.0%), piperacillin (84.4%), tetracycline (75.6%), cefuroxime (71.1%), ceftriaxone (68.9%), trimethoprim-sulfamethoxazole (65.6%), ciprofloxacin (64.4%), levofloxacin (63.3%) and cefotaxime (62.2%). On the contrary, resistance rates were low to carbapenems (< 5.0%), amikacin (5.6%), piperacillin-tazobactam (7.8%). The percentage of isolates resistant to most of the antimicrobial agents was significantly higher in ESBLs producers than that in non-producers (*p*< .0001). Notably, the low rate of resistance to carbapenems, piperacillin-tazobactam and amikacin and high rate of resistance to tetracycline and trimethoprim-sulfamethoxazole in the two groups did not differ significantly (*p*> 0.05). ESBLs producers were susceptible to carbapenems, amikacin andpiperacillin-tazobactam. These findings were indicated in Table 2.

**Table 2:** Antimicrobial Resistance ofNinety*E*. *coli*isolates from Bloodstream Infections.

Antimicrobial agents	Number of isolates (%)			*p*
	Total (n = 90)	ESBL(n = 61) ^a^	non-ESBL (n = 29)	
ampicillin	81 (90.0)	59 (96.7)	22 (75.9)	.0068
piperacillin	76 (84.4)	61 (100.0)	15 (51.7)	< .0001
ampicillin-sulbactam	46 (51.1)	38 (62.3)	8(27.6)	.0021
piperacillin-tazobactam	7 (7.8)	5 (8.2)	2(6.9)	1.0000
ceftriaxone	62 (68.9)	60 (98.4)	2(6.9)	< .0001
cefuroxime	64 (71.1)	60 (98.4)	4(13.8)	< .0001
cefepime	35 (38.9)	34 (55.7)	1(3.4)	< .0001
cefotaxime	56 (62.2)	54 (88.5)	2(6.9)	< .0001
ceftazidime	45 (50.0)	43 (70.5)	2(6.9)	< .0001
aztreonam	53 (58.9)	51 (83.6)	2(6.9)	< .0001
doripenem	0 (0)	0 (0)	0(0)	/
imipenem	0 (0)	0 (0)	0(0)	/
meropenem	2 (2.2)	2 (3.3)	0(0)	1.0000
ertapenem	4 (4.4)	4 (6.6)	0(0)	.3879
amikacin	5 (5.6)	4 (6.6)	1(3.4)	.9129
gentamicin	47 (52.2)	36 (59.0)	11(37.9)	.0613
tetracycline	68 (75.6)	48 (78.7)	20(69.0)	.3158
ciprofloxacin	58 (64.4)	50 (82.0)	8(27.6)	< .0001
levofloxacin	57 (63.3)	50 (82.0)	7(24.1)	< .0001
trimethoprim-sulfamethoxazole	59 (65.6)	43 (70.5)	16(55.2)	.1529

a ESBL(n = 61), if the ESBL confirmatory test was positive, we defined the isolate as a ESBL-producer, and the negative as a non-producer.

### Resistance Genes and Phylogenetic Groups

Sixty-one isolates were positive in the confirmatory test for ESBL. Of the 61 ESBL-producing isolates, CTX-M enzymes (57, 93.4%) predominated, with CTX-M-15(25, 43.9%), CTX-M-14 (18, 31.6%), and CTX-M-55 (9, 15.8%) being the most common types (Table 3). Some of the isolates also produced TEM-1 and OXA-1 in addition to CTX-M β-lactamases, as presented in Table 4.

**Table 3:** ESBLGenes inSixty-one ESBL positive *E*. *coli* isolates from Bloodstream Infections withinThree Hospitals.

types of ESBL		Numbers of isolates (%)				
		Total	HA^a^	HB^b^	HC^c^	*p*
ESBL		61(67.8)	21(70.0)	16(53.3)	24(80.0)	0.0827
CTX-M		57(93.4)	17(81.0)	16(100.0)	24(100.0)	0.0150
	CTX-M-14	18(31.6)	7(41.2)	7(43.8)	4(16.7)	0.1829
	CTX-M-15	25(43.9)	6(35.3)	7(43.8)	12(50.0)	0.3337
	CTX-M-55	9(15.8)	3(17.6)	2(12.5)	4(16.7)	1.0000
	CTX-M-3	1 (1.64)	0 (0.0)	0 (0.0)	1 (4.17)	/
	CTX-M-24	1 (1.64)	0 (0.0)	0 (0.0)	1 (4.17)	/
	CTX-M-27	1 (1.64)	0 (0.0)	0 (0.0)	1 (4.17)	/
	CTX-M-65	1 (1.64)	0 (0.0)	0 (0.0)	1 (4.17)	/
	CTX-M-79	1 (1.64)	1 (4.76)	0 (0.0)	0 (0.0)	/

a HA, Hospital A

b HB, Hospital B

c HC, Hospital C.

**Table 4:** Genotypes in MLST of Ninety*E*. *coli* isolates from Bloodstream Infections.

ST	Total number	phylogenetic groups of isolates (number)					
		non-ESBL	ESBL				
			N^a^	CTX-M-14	CTX-M-15	CTX-M-55	others
ST131	20	B2 (2)	B2 (17)	6	10		CTX-M-3 (1)
			A (1)				
ST405	8	D (1)	D (6)	2	2	2	
			B2 (1)		1		
ST69	5	D (4)	D (1)				
ST38	4	D (1)	D (3)	3			
ST648	4	B2 (1)	D (3)	1	2		
ST617	2	A (1)	A (1)	1			
ST10	1		B1 (1)			1	
ST44	1		B1 (1)		1		
ST167	1		A (1)	1			
ST218	1	B2 (1)					
ST95	3	B2 (1)	B2 (2)		1	1	
ST23	2		A (2)		2		
ST410	1		A (1)		1		
ST393	2		D (2)		1		
ST73	2	B2 (1)	B2 (1)			1	
ST58	1		B1 (1)				CTX-M-24 (1)
ST155	1		B1 (1)		1		
ST93	1	B2 (1)					
ST373	1		B2 (1)	1			
ST602	1		D (1)	1			
ST162	1	B1 (1)					
ST2003	4	D (1)	D (3)		1	1	
ST68	1		D (1)		1		
ST450	1	A (1)					
ST697	1	B2 (1)					
ST744	1		A (1)			1	
ST746	1		B2 (1)				CTX-M-79 (1)
ST847	1	B1 (1)					
ST998	1	B2 (1)					
ST1177	1	D (1)					
ST1193	1	B2 (1)					
ST1304	1	B1 (1)					
ST1642	1		B1 (1)	1			
ST2077	1	B1 (1)					
ST2973	1		B2 (1)	1			
ST3902	1		D (1)				CTX-M-65 (1)
ST4038	1	B1 (1)					
ST4456	1	B2 (1)					
ST4704	1	B2 (1)					
ST4995	1		B2 (1)			1	
ST5004	1		B2 (1)			1	
ST5005	1	B2 (1)					
ST5006	1	D (1)					
ST5163	1		D (1)		1		
ST5164	1		D (1)				CTX-M-27 (1)
	90	29	61	18	25	9	

a N, the phylogenetic groups (total number) of ESBL- producers.

It was indicated in Table 3 that the prevalence of ESBLs had no significant difference in each hospital. Besides, the little disparity on the total proportion of CTX-M (*p*< 0.05) was due to four non-CTX-M producing isolates in HA. Two of them were TEM-1 producers and the others harbored no β-lactamases detected with ESBLs phenotype. Given CMY and DHA genes as the most prevalent plasmid-mediated AmpC β-lactamases worldwide, we performed a screening test for the genes[23] in the four isolates and found one isolate harboring CMY enzyme.

All four major phylogenetic lineages were found among the 90 EC-BSI isolates: phylogenetic group B2(39, 43.3%) predominated, followed by D (32, 35.6%), B1 (10, 11.1%) and A (9, 10.0%). Among the 61 ESBL-producers: phylogenetic group B2 (26, 42.6%) predominated, followed by D (23, 37.7%), A (7, 11.5%) and B1 (5, 8.2%). While among the 29 non-ESBL-producers: phylogenetic group B2 (13, 44.8%) predominated, followed by D (9, 31.0%), B1 (5, 17.2%), and A (2, 6.9%). The distributions of the phylogenetic groups between ESBL-producers, non-producers and all isolates were all dominated by B2 and D. In addition, the distribution from each hospital was also nearly identical. Phylogenetic group B2 was principally associated with the most common CTX-M types CTX-M-15 (12, 48.0%). CTX-M-15 isolates belonging to phylogenetic group A, B1 and D were account for 12.0%, 8.0% and 32.0% respectively. Likewise, isolates producing CTX-M-14 and CTX-M-55 belonging to phylogenetic group B2 (both 44.4%) have exceeded those to phylogenetic group D (38.9%, 33.3%). The *bla*_*TEM-1*_ and *bla*_*OXA-1*_ positive isolates also mainly belonged to phylogenetic groups B2 (18, 45.0% and 8, 50.0%) as shown in S1 Table.

### Multilocus Sequence Typing

**Fig 1:**
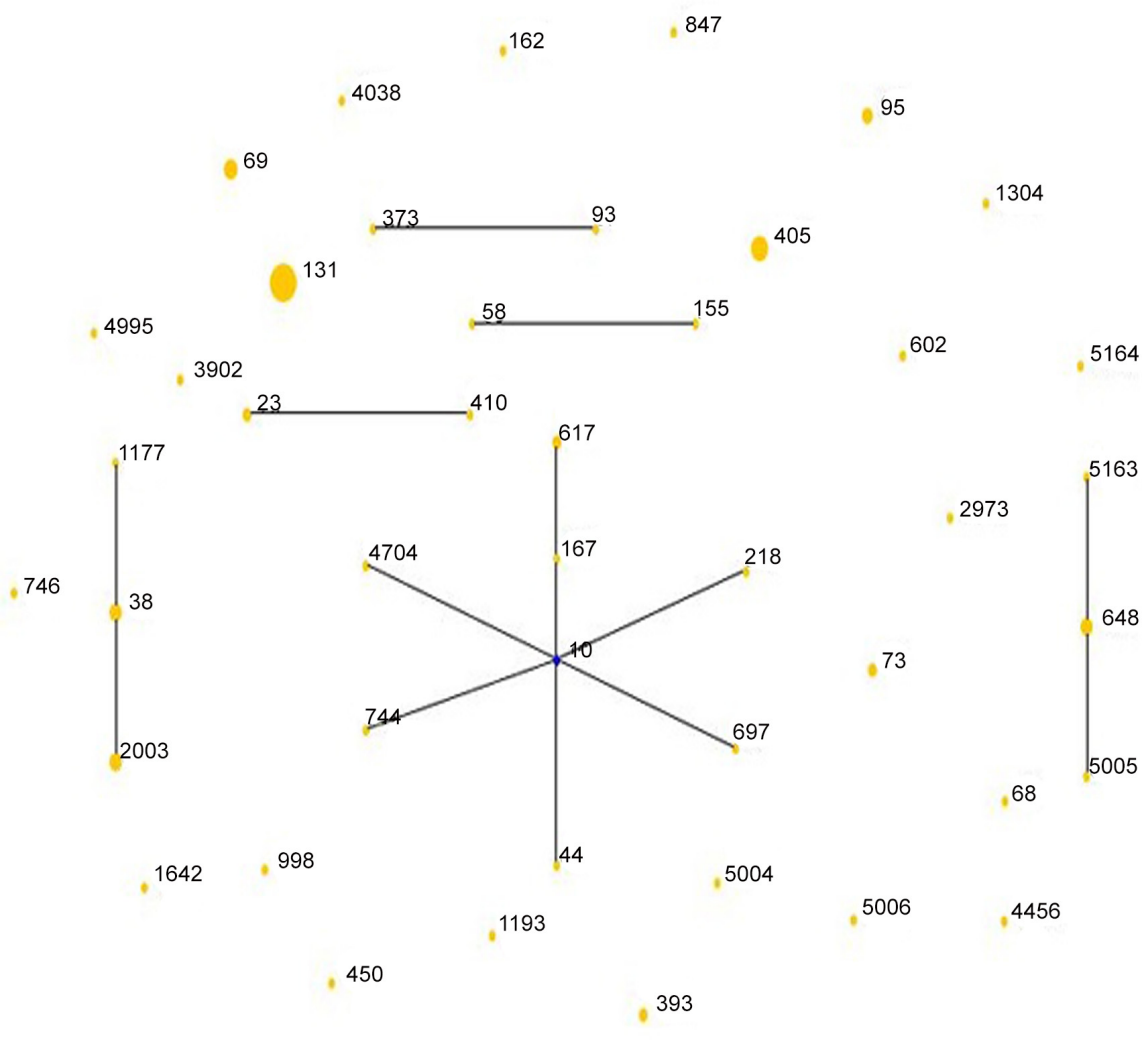
The rough sketch produced by eBURST with the stringent (default) group definition, representing ninety *E*. *coli* isolates from bloodstream infections: there were 24 singletons, 5 groups (group1: ST93, ST373; group2: ST58, ST155; group 3: ST23, ST410; group 4: ST38, ST1177, ST2003; group 5: ST648, ST5005, ST5163) and 1 clonal complex (ST10, ST167, ST617, ST218, ST697, ST44, ST744, ST4704) which was radial. The blue dot in it indicated putative founder but the line distances have no significance. The area of each yellow circle corresponded to the prevalence of the ST in the MLST data of this study.

MLST analysis distinguished 46 different sequence types (STs), which were clustered into 6 non-overlapping groups or clonal complexes and 24 singletons (Table 4, Fig 1). The sequences of the new STs has been submitted to the MLST Databases (http://mlst.warwick.ac.uk/mlst/dbs/Ecoli) and the accession numbers were ST5004, ST5005, ST5006, ST5163 and ST5164. The most frequent ST was ST131 (20/90), with the most kinds of TEM-1 (12/40), CTX-M-14 (6/18), CTX-M-15 (10/25) and OXA-1 (7/17), almost assigned to phylogenetic group B2 (19/20), followed by ST405 (8/90) which harbored the most CTX-M-55 (2/9) and mainly belonged to phylogenetic group D (7/8), and ST69 (5/90) which all belonged to phylogenetic group D. While among 61 ESBL-producers, the dominated sequence types were ST131 (18, 29.5%), ST405 (7, 11.5%), ST38 (3, 4.9%) ST648 (3, 4.9%)and ST2003(3, 4.9%). As to the distribution of main genotypes in each hospital (Table 5), B2-ST131 (phylogenetic group—sequence type) occupied the leading position, followed by D-ST405, D-ST69, D-ST38 and D-ST2003 (HA) respectively.

**Table 5:** The Distributions of Main Genotypes in Three Hospitals.

ST (number)	Hospitals (number)	phylogenetic groups of isolates (number)					
		non-ESBL	ESBL				
			N^a^	CTX-M-14	CTX-M-15	CTX-M-55	others
ST131 (20)	HA (6)		B2 (5)	4	1		
			A (1)				
	HB (6)	B2 (1)	B2 (5)	2	3		
	HC (8)	B2 (1)	B2 (7)		6		CTX-M-3 (1)
ST405 (8)	HA (1)		D (1)	1			
	HB (2)		D (2)		1	1	
	HC (5)	D (1)	D (3)	1		1	
			B2 (1)		1		
ST69 (5)	HA (2)	D (1)	D (1)				
	HB (3)	D (3)					
ST38 (4)	HB (2)		D (2)	2			
	HC (2)	D (1)	D (1)	1			
ST648 (4)	HA (1)		D (1)	1			
	HB (2)	B2 (1)	D (1)		1		
	HC (1)		D (1)		1		
ST2003 (4)	HA (3)		D (3)		1	1	
	HB (1)	D (1)					
ST95 (3)	HB (1)	B2 (1)					
	HC (2)		B2 (2)		1	1	

a N, the phylogenetic groups (total number) of ESBL- producers.

## Discussion

Since the turn of the 21st century, several worldwide reports have shown the substantial increase of ESBL-producing EC-BSI[24]. The former study of Zhao [15]was designed to characterize antimicrobial susceptibility, distribution of drug resistance genes, phylogenicity and sequence types of EC-BSI in only one hospital in Shanghai. This study expanded the project scope within three hospitals to investigate antimicrobial susceptibility and molecular epidemiology in different settings, and whether there was a spread between hospitals. Our study showed that *E*. *coli* was the most frequent microorganism isolated from blood cultures in the three hospitals, and the incidence of ESBL-producing isolates was 67.8%, similar to Zhao’s findings (62.5%)(*p* = 0.4222)[15], but much higher than that of Mexico (48.4%), Chile (23.8%), Argentina (18.1%), and Brazil (12.8%)[5]. No isolates harboring carbapenemases were found.

In consideration of the high resistance rates to some routine agents in Table 2, such as penicillins, second-generation cephalosporins, tetracyclines, fluoroquinolones and folate pathway inhibitors, which are commonly used in most hospitals in our area, we suggested TZP, AK and carbapenems as choices forempiric therapy instead. Notably, there were still 2 isolates resistant to meropenem and 4 to ertapenem (Table 2); while interestingly, these resistant isolates were all ESBL-producers, mainly CTX-M-15 producers (3/6). Considering no carbapenemases were detected in our study, the possible reason for the resistance may be porin loss combined with the production of ESBLs or AmpC β-lactamases[25]. Previous studies have confirmed the combination can confer carbapenem resistance in clinical isolates[25, 26].

Unlike other studies in China, we found that CTX-M-15 was the dominant ESBL rather than CTX-M-14, and the distribution of CTX-M enzymes in three hospitals was not statistically significant. However, the distribution of CTX-M in former study was just the opposite that CTX-M-14 was the most prevalent CTX-M enzymes in HA (i.e. Shanghai Ruijin Hospital) during 2011–2013[15]; and it was similar to the distribution of only HA in this study as well. These data demonstrated that CTX-M-types producing *E*. *coli* may exhibit geographic difference due to the various modes of transmission. In addition, isolates producing OXA-1(17/61) also accounted for a larger proportion among the β-lactamases-producers, but only found in HB and HC mainly belonging to B2-ST131, which may also be verification about different distribution of resistance genes in different settings. Furthermore, there were two isolates with ESBL phenotype not detected ESBLs, most likely due to overexpression of TEM β-lactamases and to expression of AmpC β-lactamase or the mutations of AmpC (without a coexpressed ESBL enzymes)[27]. In order to support our hypothesis, we amplify CMY and DHA genes (the most prevalent plasmid-mediated AmpC worldwide) in the possible isolates and found one isolate harboring CMY enzyme, which can denote the possible existence of AmpC β-lactamases.

MLST, an unambiguous procedure to genotype specific bacterial isolates, is a powerful tool for long-term surveillance of widespread international clones and evolutionary studies to present common ancestry lineages among bacteria[19]. Our study has demonstrated a predominance of ST131 (20/90). Differing from other sequence types, most of the ST131 isolates (>60%) were resistant to penicillins, second-generation cephalosporins, tetracyclines and fluoroquinolones. It was the most antimicrobial resistant sequence type in our study. Over the last 10 years, an extensive review and several researches have revealed CTX-M-15 as the most common ESBL of *E*. *coli* ST131 isolates[28–30], as also showed in our results (Table 4). Moreover, almost all of the ST131 *E*. *coli* belonged to phylogenetic group B2, which meant these isolates virulent[22]. Johnson et al. have concluded that the combination of antimicrobial resistance and virulence of ST131 may account for its successful epidemiologic expansion [30]. These findings were exactly supported by the molecular epidemiology results of this study, and should arouse the attention of hospitals on these isolates. Interestingly, B2-ST131 isolates harboring different types of CTX-M (CTX-M-15, 10/18; CTX-M-14, 6/18 and CTX-M-3, 1/18) in different hospitals (Table 5) confirmed the association of distinct CTX-M-types with different settings again. Table 4 presented D-ST405 as the next most prevalent group (7/90) in our study. All of the 7 D-ST405 isolates predominantly harbouredCTX-14 and CTX-M-15. In Canada [6] and Japan [31], D-ST405 was also the second most prevalent (both 7%) with CTX-M-15 and CTX-M-14 the most frequent ESBLs. These results inferred that CTX-M-14- and CTX-M-15-producing D-ST405 isolates may comprise another pandemic clonal group. It was noteworthy that among the 6 isolates resistant to carbapenems, there were 3 D-ST405 *E*. *coli* resistant to ertapenem. The study in Taiwan has reported ertapenem the least active carbapenems against the loss of porins[26], which also proved the loss of porins may account for the resistance to carbapenems. Former studies have reported the global spread of *E*. *coli* ST405 isolates with various types of CTX-M, which may be associated with NDM β-lactamase[32, 33]. We will keep tracking the production of carbapenemases in these isolates. D-ST69 was the third most prevalent group (5/90). We only identified 1 ESBL-producing D-ST69 isolate, and the low rate resembled that in the Japanese study [31]. D-ST69 isolates have never been reported as an ESBL producer[31, 34], and were usually detected from animals and environment in Europe[34]. These isolates in our study exhibited high resistance to SXT and TE (100%) but were susceptible to fluoroquinolones, which was consistent with European studies[34].

Our study suggested that, EC-BSI did not evolve from a unique ancestral background with only 31 strains (34.4%) assigned to 6 clonal groups (Fig 1). Fortunately, ST131, the most frequent sequence type, was just a singleton in this study. ST10 was the primary founder of the biggest clonal group, and we observed the ST10 clonal group consisting of 8 different STs from different locations. The ST10 clonal complex with CTX-M-14 or -15 have previously been reported in clinical isolates from Egypt and Spain, as well as in poultry from Spain[6].

In China, many hospitals don’t save clinical isolates routinely or regularly, which led to the main limitation in our study: the results may not be generalizable enough, and further studies are needed to evaluate more hospitals to determine the more actual characteristics of *E*. *coli* BSI isolates in Shanghai, even in China.

## Conclusions

In summary, prudent and rational uses of antimicrobial agents were on request according to the high rates to ampicillin (90.0%), tetracycline (75.6%), Cefuroxime sodium (71.1%) and so on. The B2-ST131 and D-ST405 clonal groups contributed to the spread of EC-BSI in Shanghai. Phylogenetic analysis indicated genetic diversity among EC-BSI. The results highlighted the association of distinct ESBLs and sequence types with different geographic areas and settings; we urgently demand more hospitals participating in the well-designed molecular epidemiological studies for more comprehensive and reliable epidemiological results. This will provide insight into the emergence and spread of EC-BSI in the three hospitals to prevent further dissemination.

## Supporting Information

S1 Table Clinical data, rates of drugresistance and molecular characteristics of 90 *E*. *coli* from bloodstream infections in three hospitals. (XLSX)
